# Comparison of Prostate-Specific Antigen Response in Black Patients with Metastatic Castration-Sensitive Prostate Cancer Initiated on Apalutamide vs Abiraterone Acetate

**DOI:** 10.36469/001c.151273

**Published:** 2025-12-29

**Authors:** Gordon Brown, Sabree Burbage, Ibrahim Khilfeh, Carmine Rossi, Shawn Du, Frederic Kinkead, Lilian Diaz, Dominic Pilon, Benjamin Lowentritt

**Affiliations:** 1 New Jersey Urology, Cherry Hill, New Jersey, USA; 2 Johnson & Johnson, Horsham, Pennsylvania, USA; 3 Analysis Group, Inc., Montréal, Québec, Canada; 4 Chesapeake Urology, Towson, Maryland, USA

**Keywords:** prostate-specific antigen, prostatic neoplasms, treatment effectiveness, real-world, androgen receptor antagonists

## Abstract

**Background:**

Improved clinical outcomes have been observed in patients with metastatic castration-sensitive prostate cancer (mCSPC) who experience deep prostate-specific antigen (PSA) responses after treatment with androgen receptor pathway inhibitors (ARPIs).

**Objective:**

This retrospective longitudinal study aimed to compare real-world PSA90 response (≥90% reduction from pretreatment levels) in Black patients with mCSPC treated with apalutamide vs abiraterone acetate.

**Methods:**

Electronic medical record (EMR) data from Precision Point Specialty Analytics were linked to claims data from the Komodo Research Database. Black adult patients with mCSPC who initiated apalutamide or abiraterone acetate on or after 9/17/2019 were included. Inverse probability of treatment weighting was used to balance patient characteristics between treatment cohorts. PSA90 was evaluated during the on-treatment period, defined as the time from index date to the earliest of treatment discontinuation, initiation of a new ARPI or radiopharmaceutical, or end of insurance claims activity or clinical activity. Weighted Kaplan-Meier curves and hazard ratios (HRs) were used to compare PSA90 responses between treatment cohorts.

**Results:**

This study included 363 patients, of which 236 initiated apalutamide and 127 initiated abiraterone acetate. At 6 months following treatment initiation, a greater proportion of patients treated with apalutamide (65.4%) vs abiraterone acetate (49.0%) achieved a PSA90 response. Patients who initiated apalutamide vs abiraterone acetate were 66% more likely to achieve a PSA90 response within 6 months of treatment initiation (HR, 1.66; 95% confidence interval, 1.18-2.35; *P* = .004). The median time-to-PSA90 response was approximately 6 months earlier for patients treated with apalutamide (3.3 months) compared with abiraterone acetate (9.1 months).

**Discussion:**

This study leveraged robust information from combined insurance claims and routinely collected EMR data to evaluate PSA90, a clinically relevant biomarker of treatment response, among Black patients with mCSPC. These results are among the first in this understudied patient population and suggest that a deeper and earlier PSA response achieved with apalutamide relative to abiraterone acetate can extend to Black patients in a real-world US clinical setting.

**Conclusion:**

Black patients treated with apalutamide experienced significantly higher PSA90 response rates than those treated with abiraterone acetate, suggesting possible clinical benefits from early treatment response in this population.

## BACKGROUND

Prostate cancer (PC) is the fifth-leading cause of cancer-related mortality among men worldwide and, in the United States, is expected to account for an estimated 313 780 new cancer cases and 35 770 deaths in 2025.^1,2^ Previously, the standard of care for metastatic castration-sensitive PC (mCSPC), also referred to as metastatic hormone-sensitive PC (mHSPC), was androgen deprivation therapy (ADT); however, the recent development of androgen receptor pathway inhibitors (ARPIs), used in combination with ADT, has provided a superior treatment option.^3^ In two phase III, randomized, placebo-controlled trials, patients with mCSPC treated with apalutamide plus ADT (TITAN) or abiraterone acetate and prednisone plus ADT (LATITUDE) experienced greater radiographic progression-free survival (rPFS) compared with patients treated with ADT and placebo.^4,5^ Results from these trials led to the US Food and Drug Administration approvals of apalutamide for mCSPC in September 2019 and abiraterone acetate for high-risk mCSPC in February 2018.^6,7^

Among Black men, persistent disparities in cancer screening, access to treatment, and economic inequalities, which, along with earlier and more aggressive disease presentation, have led to higher PC incidence and mortality rates compared with White men.^8,9^ There is limited evidence on ARPI treatment outcomes among Black patients with mCSPC, despite the need for effective therapies among this patient population. Indeed, in the pivotal TITAN trial, fewer than 2% of patients were Black men.^4^ PC treatment response can be evaluated by measuring prostate-specific antigen (PSA) levels, with an earlier and deeper PSA response associated with superior long-term outcomes in post-hoc analyses of phase III clinical trials.^10,11^ Importantly, PSA90 (ie, ≥90% reduction in PSA from pretreatment levels) is a clinically relevant early indicator of treatment success, with real-world data linking PSA90 achievement to improved disease progression outcomes following ARPI initiation, including significantly greater overall survival and a lower likelihood of progression to castration resistance.^12^ However, only one previous real-world study has evaluated PSA responses among Black patients with mCSPC following ARPI treatment, finding that approximately 75% of patients attained a PSA90 response with apalutamide treatment.^13^ Therefore, the aim of this real-world study was to compare PSA90 responses in Black patients with mCSPC treated with either apalutamide or abiraterone acetate in US clinical practice.

## METHODS

### Data Source

Data were obtained from the Precision Point Specialty (PPS) Analytics database, composed of routinely collected electronic medical record (EMR) data from multiple private, US community urology practices (February 1, 2017–December 31, 2023). PPS EMR data include demographic, clinical, and PC-specific variables that capture information on castration resistance, sites of metastases, laboratory test values (eg, PSA testing, Gleason score, testosterone), and ARPI dispensing information (eg, dosage, fill dates, amount dispensed).

To supplement EMR data from PPS, insurance claims from the Komodo Research Database (KRD) were linked by Datavant using patent-pending de-identification technology, whereby patient information was supplemented with an encrypted token that cannot be reversed to reveal the original information. KRD includes data for over 330 million US patients covered by commercial insurance, Medicaid, or Medicare (January 1, 2016–December 31, 2023). These records contain inpatient and outpatient diagnosis and procedure information, prescription fills, and billing and reimbursement data. Furthermore, KRD also includes self-reported race that was supplemented with modeling information and benchmarked with 2020 census data. All data were Health Insurance Portability and Accountability Act (HIPAA) complaint, and approval by an institutional review board was therefore not required.

### Study Design

**Figure 1.**
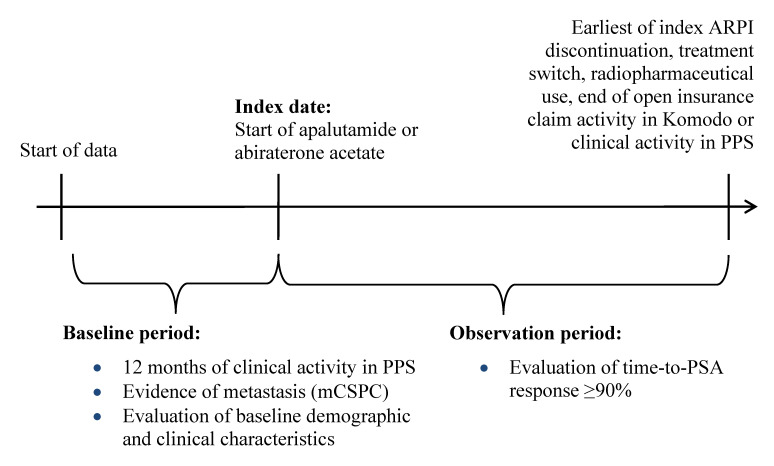
Study Design Abbreviations: ARPI, androgen receptor pathway inhibitor; mCSPC, metastatic castration-sensitive prostate cancer; PPS, Precision Point Specialty; PSA, prostate-specific antigen.

This study used a retrospective longitudinal cohort design. Patients were assigned to mutually exclusive treatment cohorts (ie, apalutamide, abiraterone acetate). The index date was defined as the first record for apalutamide or abiraterone acetate, identified as the earlier of the first in-office dispensing in PPS or the first paid pharmacy claim in KRD on or after September 17, 2019 (ie, later date of US FDA approval for apalutamide or abiraterone acetate for mCSPC^6,7^) (**Figure 1**). The baseline period comprised the 12 months prior to the index date, while the observation period spanned from the index date until the earliest of (i) index treatment discontinuation (ie, a 90-day treatment gap), (ii) the initiation of a new ARPI (ie, treatment switch, excluding first-generation androgen inhibitors), (iii) initiation of a radiopharmaceutical, or (iv) end of open insurance claim activity in KRD or clinical activity in PPS (ie, December 31, 2023).

### Sample Selection

Black adult patients with at least 1 paid pharmacy claim or dispensation for apalutamide or abiraterone acetate, at least 1 PSA measurement within the 13 weeks up to and including the index date, confirmed metastasis prior to or on the index date, and at least 12 months of clinical activity in PPS pre-index were included in this study. Exclusion criteria included patients who initiated an index ARPI prior to September 17, 2019, had a prescription for an ARPI besides the index ARPI prior to or on the index date, had evidence of castration resistance prior to or on the index date, or used radiopharmaceuticals prior to or on the index date. Castration resistance was assessed using a previously described algorithm based on clinical indicators (**Supplementary Table S1**).^14^ Metastatic disease was identified based on diagnoses codes in KRD or PPS, or a clinical indicator in PPS, for bone, nodal, or visceral metastasis on or prior to the index date. Patients were not required to have concurrent ADT use for study inclusion. In the abiraterone acetate cohort, concurrent prednisone use was not required for study inclusion.

### Study Measures and Outcomes

The primary study outcome was the proportion of patients who achieved at least a 90% reduction in PSA (ie, PSA90 response) based on the baseline PSA measurement. Baseline PSA was defined as the most recent value recorded within the 13 weeks prior to and including the index date. A PSA90 response was defined as a follow-up PSA measurement at least 90% smaller than the baseline PSA while on the index ARPI within 6 months of treatment initiation. Exploratory outcomes included the median time-to-PSA90 response and the proportion of patients who achieved a PSA90 response over the entire on-treatment observation period.

Baseline demographic characteristics (ie, age, race, index year, payer type, geographic region) and clinical characteristics (ie, time between initial metastasis diagnosis and index date, time between initial PC diagnosis and index date, metastasis type, prior ADT use, prior chemotherapy use, prior first-generation ARPI use, baseline PSA level, baseline testosterone level, initial Gleason score) were described. Post-index PSA measurement patterns were also evaluated during the observation period.

### Statistical Analyses

The study’s null hypothesis was that there was no difference in PSA90 response rates between ARPI-naïve patients treated with apalutamide vs abiraterone acetate by 6 months after treatment initiation. The alternate hypothesis was that there was a difference in PSA90 response rates between ARPI-naïve patients treated with apalutamide vs abiraterone acetate by 6 months after treatment initiation. Inverse probability of treatment weighting was used to balance potential confounding factors between patients treated with either apalutamide or abiraterone acetate. Probability estimates from logistic regression models were used to generate propensity scores (PS) based on the binary-dependent variable of apalutamide vs abiraterone acetate initiation. The estimates resulted from the logistic regression models included the following independent variables: age, index year, payer type, geographic region, time between metastasis and the index date, metastasis type (ie, visceral, bone, or nodal), time between initial PC diagnosis and the index date, de novo PC, previous chemotherapy use, previous ADT use, previous first-generation ARPI use, most recent baseline testosterone level, most recent baseline PSA level, and earliest Gleason score. A weight of 1/PS and 1/(1-PS) was attributed to patients in the apalutamide or abiraterone acetate cohort, respectively, with weights normalized using the mean weight of each respective cohort. Weights were truncated at the 95th percentile to reduce the impact of extreme weights. No patients were excluded due to weight truncation. Baseline characteristics were considered balanced if standardized differences were less than 10% between the apalutamide and abiraterone acetate cohorts after inverse probability of treatment weighting.^15^

Weighted Kaplan-Meier analyses were used to describe the proportion of patients treated with apalutamide and abiraterone acetate who achieved a PSA90 response by 6 months post-index. Weighted Cox proportional hazards models were used to calculate hazard ratio (HR) and 95% confidence interval (CI) to assess the association between index treatment and PSA90 response by 6 months (primary outcome) and over the entire observation period (exploratory outcome). All study analyses were performed using SAS Enterprise Guide software version 7.1 (SAS Institute).

## RESULTS

### Study Sample

**Figure 2.**
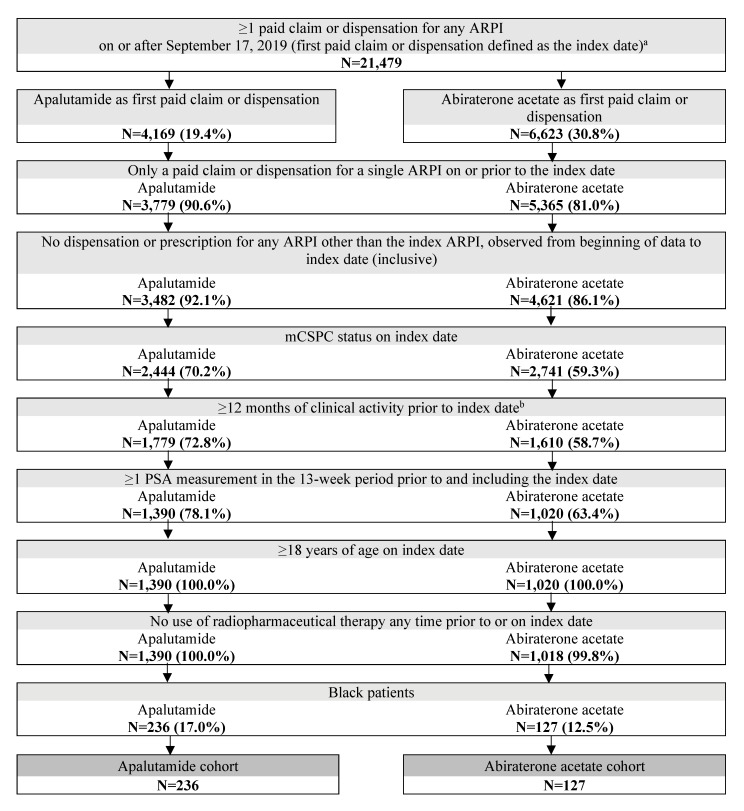
Patient Identification Flowchart Abbreviations: ARPI, androgen receptor pathway inhibitor; mCSPC, metastatic castration-sensitive prostate cancer; PSA, prostate-specific antigen. **Notes**: The US Food and Drug Administration approved apalutamide as treatment for mCSPC on September 17, 2019. Clinical activity was defined as the period from the first to the last record in the Precision Point Specialty Analytics electronic health record database.

A total of 363 patients were included in the study, of which 236 were treated with apalutamide and 127 were treated with abiraterone acetate (**Figure 2**).

### Baseline Characteristics

After weighting, the majority of baseline demographic and clinical characteristics were balanced (ie, standardized difference <10%) between patients treated with apalutamide or abiraterone acetate. The mean age of patients in the apalutamide cohort was 70.1 years, compared with 69.2 years for patients in the abiraterone acetate cohort (**Table 1**). Most patients had insurance coverage via Medicare (apalutamide, 70.7%; abiraterone acetate, 67.1%) and were from the South region (apalutamide, 71.7%; abiraterone acetate, 70.4%). The most recent mean baseline PSA level was similar between patients treated with either apalutamide (23.8 μg/L) or abiraterone acetate (24.1 μg/L). The median time from metastasis diagnosis to index ARPI initiation was 3.1 months in patients treated with apalutamide and 4.0 months in patients treated with abiraterone acetate.

**Table 1.** Baseline Characteristics for Black Patients with mCSPC

	**Non-Weighted Population**			**IPTW Population^a^**		
**Apalutamide (N = 236)**	**Abiraterone Acetate (N = 127)**	**Standardized Difference (%)**	**Apalutamide (N = 236)**	**Abiraterone Acetate (N = 127)**	**Standardized Difference (%)**	
Age, y, mean ± SD [median]	70.4 ± 8.6 [70.0]	69.0 ± 9.2 [68.0]	15.4	70.1 ± 8.8 [69.0]	69.2 ± 8.9 [68.0]	9.5
Age group, n (%)						
≤60 y	32 (13.6)	22 (17.3)	10.4	36 (15.2)	20 (16.1)	2.4
61-70 y	91 (38.6)	52 (40.9)	4.9	92 (38.9)	51 (40.5)	3.2
71-80 y	83 (35.2)	35 (27.6)	16.5	76 (32.4)	37 (29.4)	6.4
≥81 y	30 (12.7)	18 (14.2)	4.3	32 (13.5)	18 (14.0)	1.6
Geographic region,^b^ n (%)						
South	170 (72.0)	86 (67.7)	9.4	169 (71.7)	89 (70.4)	2.8
Midwest	45 (19.1)	22 (17.3)	4.5	42 (17.8)	21 (16.6)	3.0
Northeast	19 (8.1)	12 (9.4)	5.0	22 (9.3)	13 (10.3)	3.2
West	2 (0.8)	7 (5.5)	26.8	3 (1.3)	3 (2.7)	10.4
Payer type, n (%)						
Medicare	177 (75.0)	80 (63.0)	26.2	167 (70.7)	85 (67.1)	7.7
Commercial	49 (20.8)	40 (31.5)	24.6	59 (24.9)	34 (26.8)	4.4
Medicaid	9 (3.8)	4 (3.1)	3.6	10 (4.1)	6 (4.6)	2.5
Unknown	1 (0.4)	3 (2.4)	0.0	1 (0.3)	2 (1.5)	0.0
Index year, n (%)						
2019-2020	49 (20.8)	30 (23.6)	6.9	50 (21.2)	26 (20.7)	1.1
2021	49 (20.8)	28 (22.0)	3.1	49 (20.6)	25 (20.1)	1.2
2022	78 (33.1)	30 (23.6)	21.0	72 (30.7)	37 (29.2)	3.3
2023	60 (25.4)	39 (30.7)	11.8	65 (27.6)	38 (30.0)	5.3
Time between metastasis and index date, months, mean ± SD [median]	9.0 ± 17.3 [3.0]	10.6 ± 15.8 [4.1]	9.9	10.0 ± 19.7 [3.1]	10.6 ± 16.0 [4.0]	3.8
Time between PC diagnosis and index date, months, mean ± SD [median]	50.9 ± 45.3 [45.1]	49.1 ± 51.2 [30.2]	3.7	51.0 ± 46.0 [45.0]	51.3 ± 52.8 [31.4]	0.7
Metastasis type,^c^ n (%)						
Bone	150 (63.6)	64 (50.4)	26.8	140 (59.3)	69 (54.7)	9.1
Nodal	134 (56.8)	82 (64.6)	16.0	140 (59.3)	79 (62.6)	6.3
Visceral	38 (16.1)	15 (11.8)	12.4	34 (14.4)	15 (11.6)	8.4
De novo metastatic PC,^d^ n (%)	85 (36.0)	56 (44.1)	16.5	90 (38.2)	51 (40.2)	4.0
Concurrent use of ADT with index ARPI,^e^ n (%)	224 (94.9)	116 (91.3)	14.2	223 (94.6)	117 (92.4)	9.0
Prior use of ADT,^f^ n (%)	214 (90.7)	110 (86.6)	12.8	209 (88.7)	110 (86.6)	6.6
Prior use of first-generation ARPI,^g^ n (%)	37 (15.7)	16 (12.6)	8.9	37 (15.7)	19 (15.3)	1.0
Prior use of chemotherapy,^h^ n (%)	3 (1.3)	3 (2.4)	8.2	4 (1.5)	2 (1.7)	2.0
Baseline PSA level,^i^ μg/L, mean ± SD [median]	22.6 ± 51.5 [3.3]	22.0 ± 54.7 [2.1]	1.3	23.8 ± 53.7 [3.3]	24.1 ± 57.5 [2.4]	0.6
Baseline testosterone tests,^j^ n (%)	176 (74.6)	92 (72.4)	4.8	172 (72.8)	92 (72.7)	0.4
Testosterone <1.735 nmol/L^k^	113 (64.2)	69 (75.0)	23.6	114 (66.4)	65 (70.9)	9.7
Initial Gleason score,^l^ n (%)						
≤6	23 (9.7)	12 (9.4)	1.0	23 (9.7)	13 (9.8)	0.3
7	73 (30.9)	33 (26.0)	11.0	70 (29.5)	36 (28.1)	3.2
8	24 (10.2)	28 (22.0)	32.7	31 (13.1)	20 (15.9)	7.9
9	49 (20.8)	23 (18.1)	6.7	47 (20.0)	24 (19.3)	1.9
10	2 (0.8)	1 (0.8)	0.7	2 (0.8)	1 (0.8)	0.4
Unknown	65 (27.5)	30 (23.6)	9.0	63 (26.7)	33 (26.1)	1.5

Abbreviations: ADT, androgen deprivation therapy; ARPI, androgen receptor pathway inhibitor; mCSPC, metastatic castration-sensitive prostate cancer; IPTW, inverse probability of treatment weighting; PC, prostate cancer; PSA, prostate-specific antigen.

^a^Of note, the number of patients reported in this weighted population represents the sum of weights for the corresponding non-weighted patients, rounded to the nearest integer. The proportions displayed were calculated prior to the rounding and may be slightly different than if they were calculated based on rounded numbers.

^b^Geographic region was defined by US census areas.

^c^Types of metastases were defined at any time prior to (and including) the index date. Types of metastases were not mutually exclusive.

^d^De novo metastatic PC was defined as ≤180 days between first observed PC diagnosis and date of metastasis.

^e^Concurrent ADT use was defined as having a claim for any ADT medication from 180 days prior to 180 days after the index date.

^f^Prior use of ADT medication was defined as any ADT administration at any time prior to (and excluding) the index date.

^g^Prior use of first-generation ARPI was defined as any prescription for bicalutamide, nilutamide, or flutamide, at any time prior to (and excluding) the index date.

^h^Prior chemotherapy use was defined as any administration at any time prior to (and excluding) the index date.

^i^Baseline PSA was evaluated as the most recent value from 13 weeks pre-index up to, and including, the index date.

^j^Testosterone testing was evaluated during the 12-month baseline period and included the index date, with the most recent value reported.

^k^Patients’ mCSPC status were evaluated using their records and baseline testosterone may not be synchronous with mCSPC designation.

^l^Gleason score was evaluated at any time prior to and including the index date.

### PSA Outcomes

**Figure 3.**
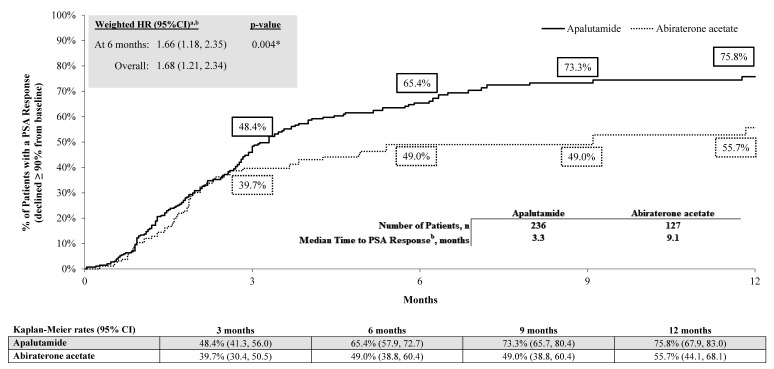
Comparison of Time-to-PSA90 Response Among Black Patients with mCSPC Abbreviations: CI, confidence interval; HR, hazard ratio; mCSPC, metastatic castration-sensitive prostate cancer; PSA, prostate-specific antigen. *Significant at the 5% level. ^a^A hazard ratio >1 indicates that the apalutamide cohort had a higher rate of PSA response ≥90% compared with the abiraterone acetate cohort. ^b^PSA90 response was defined as the first decline for a follow-up PSA value of ≥90% relative to the most recent baseline PSA value observed up to and including the index date.

The mean (median) on-treatment observation period was 11.2 (7.5) months for patients treated with apalutamide and 9.4 (6.2) months for patients treated with abiraterone acetate. A greater proportion of patients with mCSPC treated with apalutamide achieved a PSA90 response by 6 months of treatment initiation (65.4%) relative to those treated with abiraterone acetate (49.0%) (**Figure 3**). Compared with patients treated with abiraterone acetate, patients treated with apalutamide had a 66% greater likelihood of achieving a PSA90 response within 6 months of treatment initiation (HR, 1.66 [95% CI, 1.18-2.35]; *P*=.004). The median time-to-PSA90 response was also shorter among patients treated with apalutamide vs abiraterone acetate, with a median time from ARPI initiation to PSA90 response of 3.3 months for patients treated with apalutamide and 9.1 months for patients treated with abiraterone acetate.

### PSA-Related Measurements

The proportion of patients with at least 1 PSA test during the observation period was 81.9% and 79.0% for patients treated with apalutamide and those treated with abiraterone acetate, respectively (**Table 2**). The mean (median) number of PSA tests administered per year was 4.0 (3.4) for patients treated with apalutamide and 5.3 (4.1) for patients treated with abiraterone acetate. The proportion of patients with at least 1 PSA test within the first 6 months post-index was similar in patients treated with apalutamide (78.4%) and abiraterone acetate (76.5%).

**Table 2.** PSA Testing Among Black Patients with mCSPC

	**Apalutamide^a^ (N = 236)**	**Abiraterone Acetate^a^ (N = 127)**
On-treatment observation period, months, mean ± SD [median]	11.2 ±10.4 [7.5]	9.4 ±9.8 [6.2]
Patients with ≥1 PSA test, n (%)	193 (81.9)	100 (79.0)
Within 3 months of observation	164 (69.4)	88 (69.4)
Within 6 months of observation	185 (78.4)	97 (76.5)
No. of follow-up PSA tests per year, mean ± SD [median]	4.0±3.6 [3.4]	5.3±5.1 [4.1]
Patients with a PSA test on average every 3 mo, n (%)	93 (39.3)	65 (51.4)
Patients with a PSA test on average every 6 mo, n (%)	175 (74.2)	94 (74.2)

Abbreviations: PSA, prostate-specific antigen; mCSPC, metastatic castration-sensitive prostate cancer.

^a^The number of patients reported in this weighted population represents the sum of weights for the corresponding non-weighted patients, rounded to the nearest integer. The proportions displayed were calculated prior to the rounding and may be slightly different than if they were calculated based on rounded numbers.

## DISCUSSION

In this real-world analysis of Black ARPI-naïve patients with mCSPC, apalutamide was associated with a 66% higher PSA90 response rate relative to abiraterone acetate. Additionally, the median time-to-PSA90 response was achieved nearly 6 months earlier among the apalutamide cohort compared with the abiraterone acetate cohort. These results represent the first comparison of PSA90 responses after ARPI initiation in this patient population and suggest that treatment with apalutamide can result in a deeper and earlier PSA response compared with treatment with abiraterone acetate in a real-world US clinical setting.

There is a lack of published evidence comparing clinical outcomes for Black patients with mCSPC treated with apalutamide or abiraterone acetate. Two recent real-world studies of US patients with mCSPC, in which fewer than 20% of patients were Black, compared PSA90 responses between those treated with apalutamide or abiraterone acetate.^16,17^ As with the current study, both used PPS EMR data, with one also using linked insurance claims data from KRD.^17^ The present study similarly leveraged both insurance claims and routinely collected EMR data to provide a comprehensive view of patients’ clinical background, enhancing the robustness of the findings. These previous studies found that patients treated with apalutamide were 53% and 68%, respectively, more likely to achieve a PSA90 response within 6 months of treatment initiation than patients treated with abiraterone acetate. Furthermore, patients treated with apalutamide achieved a PSA90 response after approximately 3.5 months in both studies, compared with approximately 10 months or not reached for patients treated with abiraterone acetate. These findings are consistent with the observations among Black patients with mCSPC in the current study.

Additionally, the likelihood of achieving a PSA90 response within 6 months of apalutamide initiation in the current study (65.4%) was similar to the likelihoods observed in the representative populations of patients with mCSPC in the previous studies (66.2% and 63.9%),^16,17^ indicating a comparable ARPI response within this subgroup of Black patients. Previous literature has reported significantly worse survival among Black vs White patients with mCSPC.^18^ However, access-related variables were found to account for most of the excess risk of death observed in Black vs White men receiving treatment for metastatic PC. Other studies have similarly demonstrated that Black men with PC experience worse overall outcomes than White men with PC, primarily due to restricted access to care and treatment.^19–21^ Results from the current study support the effectiveness of apalutamide, as a treatment option for Black patients with mCSPC, based on early, deep PSA90 response. A recent study also highlighted how genetic testing can help assess patients risk profile to better guide treatment decisions in men of African ancestry, given recent approvals of efficacious targeted therapies for advanced PC.^22^ There exists a need for further research to disentangle the effects of race from other correlated factors, such as access to care and socioeconomic status, that may also influence treatment outcomes among patients with advanced PC.

PSA is a well-established biomarker for monitoring treatment response in advanced PC, with deeper and earlier declines linked to improved survival outcomes in both clinical trial and real-world settings.^10–12,23–27^ Notably, a recent real-world analysis found that apalutamide was associated with a 26% lower risk of death at 24 months compared with abiraterone acetate among patients with mCSPC.^28^ However, additional research is warranted to assess whether the higher PSA response rates observed with apalutamide treatment in this study ultimately lead to improved long-term outcomes in Black patients.

While Black patients with mCSPC experience increased rates of PC-related mortality,^8^ only one real-world study has characterized PSA outcomes among Black patients with mCSPC treated with an ARPI.^13^ In an analysis by Bivins et al, 76.5% of Black patients with mCSPC who received apalutamide treatment achieved a PSA90 response, with a median time to PSA90 of 2.9 months following treatment initiation.^13^ Thus, the current study provides novel real-world evidence on PSA responses in Black patients with mCSPC following treatment with two different ARPIs.

### Limitations

Limitations of this study include those inherent to the analysis of retrospective EMR and administrative claims data, such as data inaccuracies or omissions with regards to diagnosis dates, treatment start dates, or other variables, as well as possible mislinkages between the datasets. In particular, socioeconomic data were not available. As PSA response data were obtained from the PPS EMR database, any PSA testing and results from outside of the PPS network would not have been captured, potentially resulting in misclassification of the study outcome. Furthermore, if PSA testing was performed more often for one ARPI over the other, surveillance bias may have been introduced to the analyses. However, this is unlikely as patients treated with either apalutamide or abiraterone acetate received a similar mean number of follow-up PSA tests per year (apalutamide, 4.0 tests; abiraterone acetate, 5.3 tests). Regression analyses could only adjust for measured covariates, and residual confounding may be present. Finally, while a high degree of overlap was observed between patients with mCSPC in the PPS and Komodo databases, patients included in this study may not be representative of the overall Black mCSPC population in the US, limiting study generalizability.

## CONCLUSIONS

In Black patients with mCSPC, treatment with apalutamide was associated with a significantly higher PSA90 response rate within 6 months of treatment compared with treatment with abiraterone acetate. Further, Black patients with mCSPC treated with apalutamide achieved a PSA90 response approximately 6 months earlier than patients treated with abiraterone acetate. This study provides novel and valuable evidence on ARPI treatment in an understudied population of patients with mCSPC. These results are consistent with previous real-world analyses showing greater effectiveness of apalutamide compared with abiraterone acetate observed in the broader mCSPC population. Importantly, these findings demonstrate that this benefit also applies to Black patients, an understudied population in PC. Observing this continued trend underscores the importance of ensuring that clinical benefits are maintained across diverse patient populations.

### Disclosures

G.B. is an employee of New Jersey Urology and has received consulting fees from Johnson & Johnson. S.B., I.K., and S.D., are employees and stockholders of Johnson & Johnson. C.R., F.K., L.D., and D.P. are employees of Analysis Group, Inc., a consulting company that has provided paid consulting services to Johnson & Johnson. B.L. is an employee of Chesapeake Urology Associates and has received consulting fees from Johnson & Johnson.

### Ethics Statement

Data were de-identified and comply with the patient requirements of the Health Insurance Portability and Accountability Act (HIPAA) of 1996; therefore, no review by an institutional review board was required per Title 45 of CFR, Part 46.101(b)(4) (https://www.hhs.gov/ohrp/regulations-and-policy/regulations/45-cfr-46/#46.101).

### Presentations

Part of the material in this manuscript was presented at the American Society of Clinical Oncology Genitourinary Cancers (ASCO GU) Symposium, held from February 13-15, 2025, in San Francisco, California.

## Supplementary Material

Online Supplementary Material

## Data Availability

The data that support the findings of this study were used under license. These data cannot be shared as restrictions apply to their availability.
