# Supplementary material for: Comparison of Prostate-Specific Antigen Response in Black Patients with Metastatic Castration-Sensitive Prostate Cancer Initiated on Apalutamide vs Abiraterone Acetate
Source: J Health Econ Outcomes Res. 2025 Dec 29;12(2):270–8. doi: 10.36469/001c.151273 (PMC12755229; doi:10.36469/001c.151273)
Supplement: Online Supplementary Material [file jheor_2025_12_2_151273_323549.pdf]

Journal of Health Economics and Outcomes Research

Online Supplementary Material

Comparison of Prostate-Specific Antigen Response in Black Patients with Metastatic Castration-Sensitive Prostate Cancer Initiated on Apalutamide vs Abiraterone Acetate. *JHEOR*. 2025;12(2):270-278.
doi:10.36469/jheor.2025.151273

Table S1: Castration-Resistance Algorithm

This supplementary material has been provided by the authors to give readers additional information about their work.

This is an open-access article distributed under the terms of the Creative Commons Attribution 4.0 International License (CCBY-4.0). View this license's legal deed at http://creativecommons.org/licenses/by/4.0 and legal code at http://creativecommons.org/licenses/by/4.0/legalcode for more information.

Assessment of castration resistance was based on a previously published algorithm incorporating presence of androgen deprivation therapy (ADT) and prostate-specific antigen (PSA) levels.[1] All patients with evidence of castration resistance prior to or on the index date were excluded from this study. Castration resistance was defined using one of the following four criteria with the earliest observed date for any of these criteria defined as the initial castration resistance date:

Table S1. Castration-Resistance Algorithm

Castration Resistance Criteria	Description
≥1 indicator for CRPC based on provided variables in PPS	The indicator considered 1 of the following criteria:<br>• 2 consecutive rises in PSA while on continuous luteinizing hormone-releasing hormone therapy for >6 mo<br>• Presence of enzalutamide before it received a non-CRPC indication,<br>• Manual data entry by a clinical user,<br>• Presence of the Z19.2 ICD-10-CM diagnosis code<br>• Parsable encounter notes in the EMR where a physician indicated that patients progressed to CRPC<br>If a patient was considered by ≥1 of these criteria as castration-resistant, then the patient was identified as castration-resistant.
≥1 diagnosis for castration-resistant malignancy status	≥1 claim with a diagnosis code ICD-10-CM: Z19.2 identified in PPS or KRD
Surgical castration and ≥1 rise in PSA from post-castration nadir	≥1 record for bilateral orchiectomy observed at any time before the first observed ARPI used AND ≥2 PSA test results at any time before the first observed ARPI used (including 1 nadir and 1 post-nadir) after the procedure, with ≥1 rise in PSA (of ≥25% with an absolute increase of ≥2 ng/mL) after nadir. Nadir was defined as the lowest PSA value observed between the procedures and the first ARPI claim.
Medical castration and ≥1 rise in PSA from post-castration nadir	≥90 days of continuous ADT use observed at any time before the first observed ARPI use AND ≥2 PSA test results at any time before the first observed ARPI used (including 1 nadir and 1 post-nadir) within the same episode of continuous ADT, with ≥1 rise 1 PSA (of ≥25% with an absolute increase of ≥2 ng/mL) after nadir. Nadir was defined as the lowest PSA value observed between the procedures and the first ARPI claim.

Abbreviations: ADT, androgen deprivation therapy; ARPI, androgen receptor pathway inhibitor; CRPC, castration-resistant prostate cancer; EMR, electronic medical record; ICD-10-CM, *International Classification of Diseases, Tenth Revision, Clinical Modification;* KRD, Komodo Research Database; PPS, Precision Point Specialty; PSA, prostate-specific antigen.

REFERENCE

1. Freedland SJ, Ke X, Lafeuille MH, et al. Identification of patients with metastatic castration-sensitive or metastatic castration-resistant prostate cancer using administrative health claims and laboratory data. *Curr Med Res Opin*. 2021;37(4):609-622. doi:10.1080/03007995.2021.1879753
